# COVID-19 and shielding: experiences of UK patients with lupus and related diseases

**DOI:** 10.1093/rap/rkab003

**Published:** 2021-01-21

**Authors:** Melanie Sloan, Caroline Gordon, Elliott Lever, Rupert Harwood, Michael A Bosley, Mark Pilling, James Brimicombe, Felix Naughton, Moira Blane, Chanpreet Walia, David D’Cruz

**Affiliations:** 1 Department of Public Health and Primary Care, University of Cambridge School of Clinical Medicine, Cambridge; 2 Rheumatology Research Group, Institute of Inflammation and Ageing, College of Medical and Dental Science, University of Birmingham, Birmingham; 3 Rheumatology Department, Northwick Park Hospital, London; 4 Patient and Public Involvement in Lupus Research Group, Institute of Public Health, University of Cambridge, Cambridge; 5 Behavioural and Implementation Science Group, School of Health Sciences, University of East Anglia, Norwich; 6 LUPUS UK, St James House, Romford; 7 The Louise Coote Lupus unit, Guy’s and St Thomas’ NHS Foundation Trust, London, UK

**Keywords:** COVID-19, shielding, rheumatology, systemic lupus erythematosus, systemic autoimmune rheumatic disease, lupus, quality of life, patient views, patient behaviour, mixed methods

## Abstract

**Objective:**

The shielding guidance in the UK for the clinically extremely vulnerable (CEV) commenced on 23 March 2020 in response to the coronavirus disease 2019 (COVID-19) pandemic. The purpose of this study was to explore the impact of the pandemic and shielding on patients with lupus and related systemic autoimmune rheumatic diseases (SARDs).

**Methods:**

This was a mixed-methods cohort study (*n* = 111) including pre-lockdown baseline surveys (March 2020), follow-up surveys (June 2020) and in-depth interviews during July 2020 (*n* = 25).

**Results:**

Most participants had a high level of anxiety regarding their mortality risk from COVID-19 and supported the concept of shielding. Shielding allocations and communications were perceived as inconsistently applied and delivered. More than half of those not classified as CEV reported feeling abandoned, at increased risk and with no support. Shielding communications increased feelings of being ‘cared about’, but also increased fear, and the ‘vulnerable’ labelling was perceived by some to damage social and self-identity. More than 80% of those classified as CEV stated that the classification and subsequent communications had changed their social-mixing behaviour. Despite many negative impacts of COVID-19 and shielding/lockdown being identified, including isolation, fear and reduced medical care, the quantitative data during the pandemic showed increases in most measures of wellbeing (which was low at both time points) from pre-lockdown, including reductions in the impact of fatigue and pain (*P*-values < 0.001).

**Conclusion:**

Shielding classifications and communications were, in general, viewed positively, although they were perceived as inconsistently delivered and anxiety-provoking by some participants. More frequent positively framed communication and wellbeing support could benefit all SARD patients. Slower-paced lockdown lifestyles might confer health/wellbeing benefits for some people with chronic diseases.

**Key messages**

- The concept of shielding was supported by lupus/systemic autoimmune rheumatic disease patients and engendered more socially avoidant behaviour.
- Inconsistently applied shielding allocation omitted many patients, generating feelings of abandonment and endangerment.
- Multiple health/wellbeing measures improved unexpectedly during lockdown/shielding, probably from a slower-paced lifestyle.

## Introduction

At its outset, coronavirus disease 2019 (COVID-19) was anticipated to have a significant impact on the wellbeing of patients with lupus and related systemic autoimmune rheumatic diseases (SARDs). These patients have complex multi-system disease, often with unpredictable flares and frequent use of immunosuppression, the main criteria for risk assessment and shielding classification in this group [1].

The first UK ‘lockdown’, which commenced on 23 March 2020, was accompanied by more stringent government guidance to those identified as being clinically extremely vulnerable (CEV), initially by National Health Service (NHS) digital [2] and subsequently by hospital clinicians and general practitioners (GPs) for further eligible patients. The identification process and shielding guidance/timescales differed for each UK devolved nation. The CEV were advised to ‘shield’, which included not leaving homes/gardens (except for medical appointments) and physically distancing from other household members [3]. As a group, lupus patients and those with related conditions are likely to have been affected disproportionately by the negative impact of shielding on mental health (MH) [4] owing to approximately one-third meeting shielding criteria [5].

In addition, the adverse impact of other aspects of the pandemic on MH found in the UK population [6, 7] could also have impacted patients with lupus and related diseases disproportionately, in part owing to some of the identified risk factors also being associated with SARDs, including: having a pre-existing physical or mental health [8] condition and/or multimorbidity [6]; lower income and/or not having a job [9], with pre-pandemic studies having found employment and income disadvantage among lupus and SARD patients [10–12]; and reduced access during the pandemic to normal chronic illness care [13–15]. Our previous COVID-19 study found that the majority of this cohort of participants reported that disruptions to their medical care had adversely impacted their MH [14].

Although the published literature suggests the possibility of multiple negative impacts of the pandemic on SARD patients [4, 5, 13, 14], the aim of this study was to explore in depth the nature and magnitude of the negative impacts (within the limits of the sample), particularly in relationship to shielding.

## Methods

Detailed methods, including the consolidated criteria for reporting qualitative research (COREQ) checklist [16], are provided in Supplementary Data S1, available at *Rheumatology Advances in Practice* online.

### Data collection

This mixed-methods study integrates findings from: (a) quantitative and qualitative data from a cohort of participants [14] who completed both pre-lockdown baseline (4–10 March 2020) and follow-up (10–21 June 2020) online surveys; (b) a content and qualitative analysis of COVID-19 posts on the LUPUS UK forum (March–August 2020); and (c) in-depth interviews (July 2020).

The cohort was from a pre-existing longitudinal study [14] focusing on MH/wellbeing, medical care and peer support (pre-registered: ISRCTN-14966097). Informed consent was obtained before surveys and interviews. The online surveys were made available on the LUPUS UK forum and Lupus support UK Facebook group for study sign-up for any prospective participant meeting the following inclusion criteria: a diagnosis of lupus/other related SARD (as detailed on participants’ clinic letters); ≥18 years of age; and resident in the UK.

Ethical approval was obtained through the Cambridge Psychology Research Committee (PRE.2019.099: approval for original peer-support trial; PRE.2020.089: approval for COVID-19 related changes to survey and interviews; and PRE.2018.120: approval for analysis of the LUPUS UK forum).

The primary outcome measure was the Warwick-Edinburgh Mental Wellbeing Scale (WEMWS) [17], with the follow-up survey adapted to include questions on the impact of the pandemic and shielding. Survey responses were analysed and used to inform purposive sampling (non-random selection of interviewees to ensure a wide range of socio-demographics and experiences/views). Interviews explored participants’ experiences of the shielding policy, communication, medical care, and perceived impacts on MH and behaviour. Interviews were conducted by M.S., predominantly by telephone. They lasted ∼1 hour and were audio-recorded and transcribed verbatim. Interviewing continued until thematic saturation [18] (no novel findings from subsequent interviews) was reached.

### Analysis

Integration of data sources occurred throughout, with the qualitative components being analysed thematically [19] to further explore and explain quantitative results. Validity and reliability were enhanced by: M.S. coding all data (using Nvivo12), R.H. double-coding 25% of transcripts, E.L. and M.A.B. reviewing qualitative data and independently generating proposed themes, confirming that patients were in agreement with arising themes (member checking) [20], triangulation of multiple data sources and consideration of deviant cases [21]. Emerging themes were then agreed by the wider team, including patient representatives. Quantitative data were analysed using SPSS v.26 (IBM Corp., Armonk, NY). Test diagnostics were examined, and all were satisfactory. Student’s paired *t* tests were used for assessing change over time for continuous outcomes, and χ^2^ test or Fisher’s exact test for categorical outcomes.

## Results

Survey respondents (*n* = 111) and interviewees (*n* = 25) encompassed a broad range of socio-demographic characteristics, although all but two participants were female (Table 1). Fifty-one per cent of survey participants reported being allocated to the shielding category. Any percentages/statistics reported are from the surveys.

**Table 1:** Participant characteristics (*n* = 111)

Characteristic	Number (survey, *n* = 111)	Percentage (survey)	Number (interview, *n* = 25)	Percentage (interview)
Age band (years)				
20–29	20	18	5	20
30–39	18	16	3	12
40–49	23	21	6	24
50–59	31	28	5	20
60–69	15	14	4	16
70+	4	4	2	8
Diagnosis				
SLE	87	78	15	60
UCTD	7	6	4	16
SSS	5	5	2	8
MCTD or overlap CTD	6	5	1	4
Cutaneous lupus	4	4	2	8
Probable or incomplete lupus	2	2	1	4
Employment				
Employed full time	27	24	5	20
Employed part time	22	20	5	20
Self-employed	7	6	3	12
Not currently working owing to health	31	28	7	28
Retired	19	17	5	20
Other	5	5	0	0
Ethnicity				
Asian	6	5	2	8
White	100	90	19	76
Black	2	2	2	8
Mixed race	3	3	2	8
Gender				
Female	109	98	25	100
Male	2	2	0	0
Qualifications				
None	2	2	0	0
GCSE/O levels (equivalent)	19	17	4	16
A levels (or equivalent)	25	23	5	20
Degree or above	60	54	16	64
Prefer not to say	5	5	0	0
Country of residence				
England	84	76	17	68
Scotland	16	14	5	20
Wales	9	8	3	12
Northern Ireland	2	2	0	0

Adapted with permission from Sloan *et al.* [14].

Four key themes were identified: (a) impact of COVID-19 on MH/wellbeing; (b) inconsistency in risk classification and communication; (c) the impact of the risk classification and shielding on multiple domains; and (d) lessons learnt from lockdown lifestyle for SARD management.

### Theme 1: impact of COVID-19 on MH/wellbeing

The primary outcome measure of wellbeing, using changes in the total WEMBS (scale from 14 to 70, with higher scores representing better wellbeing), showed a small and non-statistically significant improvement (*P* = 0.084) from pre-lockdown (38.6) to during-pandemic (39.8) surveys, with no significant difference between shielders and non-shielders.

### Negative influences on MH

#### Isolation

The three inter-personal WEMBS wellbeing measures (feeling useful, interested in other people, and feeling close to others) all showed (non-significant, *P*-values > 0.1) small reductions during lockdown. Multiple participants discussed feeling isolated and depressed from reduced social interaction, which was especially severe among those fully following shielding guidance and living alone:

Dark moods … loneliness. Hard living on my own. Very dark days. (Participant 67, 60–69 years old)

#### Fear

Many participants estimated their mortality risk from COVID-19 as very high and expressed great anxiety during interviews and in forum conversations. Anxiety was often discussed as being increased by official correspondence identifying vulnerability ‘in black and white’ or, conversely, through not having received specific or sufficient information:

I felt afraid and quite panicked, which led to a massive flare, which lasted 6 weeks.… I don’t know if I will ever feel safe again. (Participant 132, 40–49 years old)

Non-shielders who felt they should have been allocated to the shielding group reported increased fears around potential exposure:

I really can't afford to lose my job, but I really am so scared of dying!!!… I’ve already been told by my lovely nurse I have a DNR [do not resuscitate order] on my record for COVID-19 and I will not make it through it. This I know myself; I can't fight. (Forum, 40–49 years old)

Additional risk factors, such as being from a Black, Asian and minority ethnic (BAME) group, also increased anxiety:

Did make me worry more … mainly because they still don't know for a fact why black people were dying so much … very scary and made me a lot more vigilant in being careful and safe. (Participant 136, 30–39 years old)

#### Decreased medical support

Fewer than 30% of survey respondents agreed that they had felt medically supported during the pandemic. Most reported cancellations of appointments, and some received no communications or response to requests for help from rheumatology departments:

Flaring badly … unable to get hold of my rheumatology team to help me … much more depressed than I was before the coronavirus outbreak. This is partly due to isolation from friends and family but also because I have felt very vulnerable due to my inability to get the medical support I have needed. (Survey, 40–49 years old)

### Positive influences on MH

#### Less pressure and increased parity

**Fig. 1:**
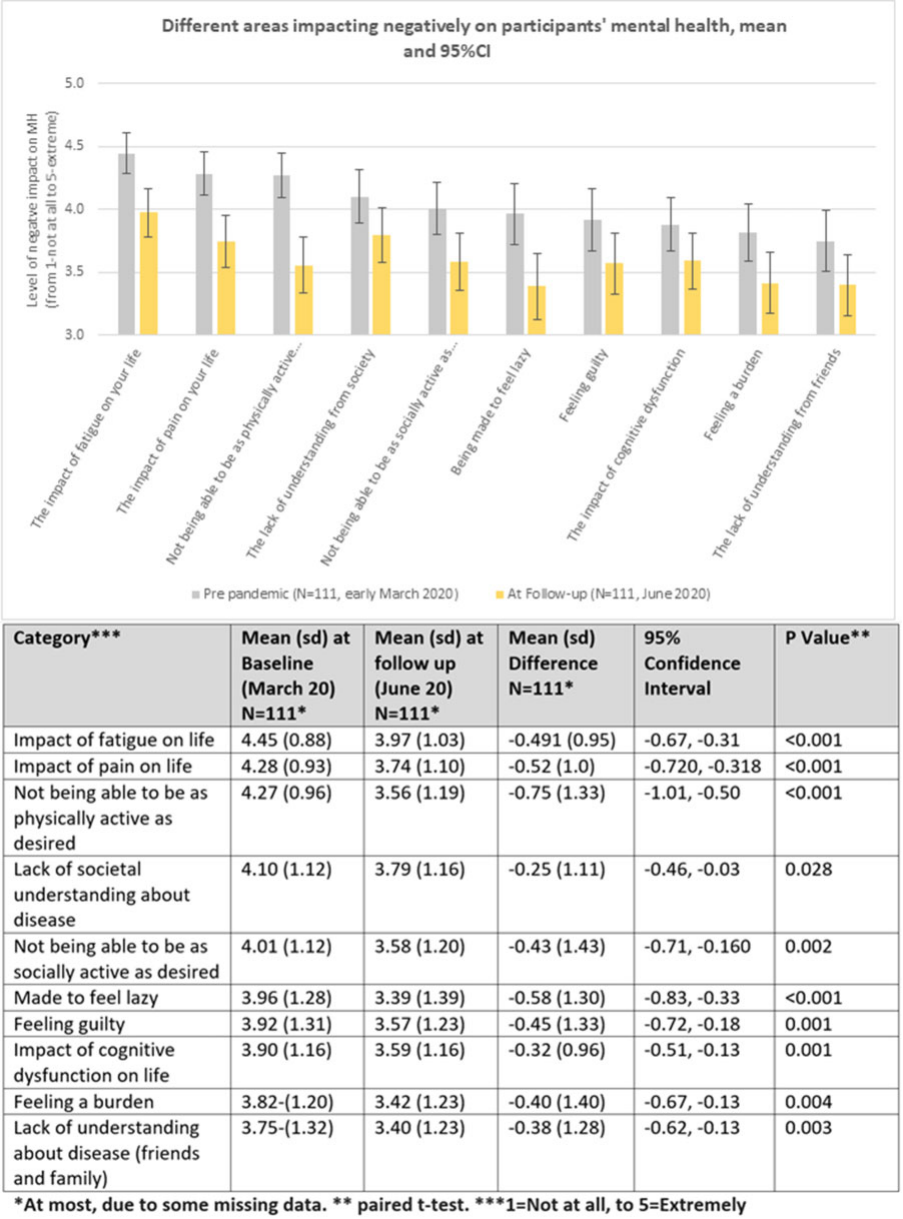
Changes to impacts on mental health from baseline (pre-lockdown, March 2020) to follow-up (June 2020)

Most WEMBS measures were higher during lockdown than pre-lockdown, with three significant improvements using Student’s paired *t* test: feeling relaxed [mean difference (MD) 0.279, 95% CI: 0.103, 0.455, *P* = 0.002], energy to spare (MD 0.198, 95% CI: 0.027, 0.0370, *P* = 0.024) and dealing with problems well (MD 0.243, 95% CI: 0.055, 0.432, *P* = 0.012). Negative impacts on MH were found to have significantly lower scores (less impact) during lockdown, as shown in Fig. 1. The largest changes were in reductions in the impact of being unable to be as physically active as desired (MD −0.752, 95% CI −1.01, −0.495. *P* < 0.001), the impact of fatigue and pain on lives, and being made to feel lazy (*P*-values < 0.001). These findings were explored during interviews, with participants surmising that a reduction in pressure of normal life combined with the rest of society being restricted in a similar manner reduced the negative impact on the chronically diseased:

In terms of the parity, so everyone is locked in so … we haven’t obviously met up, but then no-one has, so we’re all in the same boat, so I’m not missing out, which is nice. (Participant 108, 30–39 years old)

#### Resilience

The majority of participants felt they had coped better with lockdown than the general population (66% of survey participants), explained, in part, as already having heavily constrained lives pre-pandemic and/or greater resilience from living with a chronic disease:

We are a group of people who know how to deal with this. More than most. We are all fighters who know our bodies better than anyone. (Participant 55, 40–49 years old)

Fig. 1 shows the changes to impacts on mental health from baseline (pre-lockdown, March 2020) to follow-up (June 2020).

### Theme 2: inconsistency in classification and communication

#### Communication of risk group

**Fig. 2:**
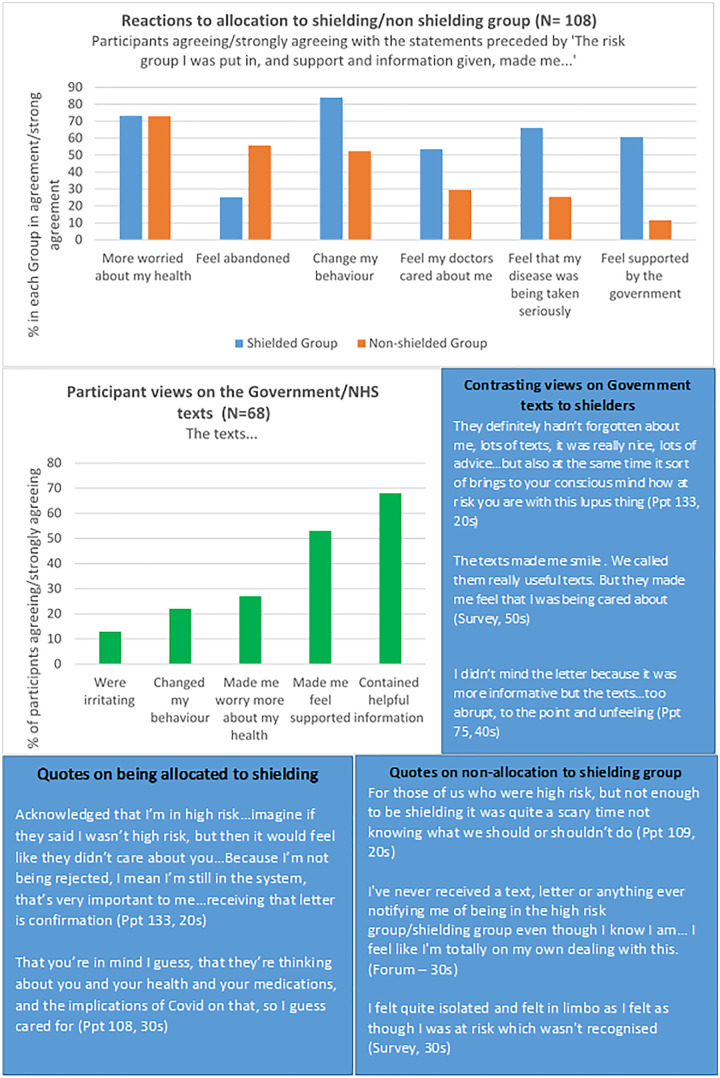
Reactions to allocation of risk group and views on government/National Health Service texts

Although some participants received prompt communication of their risk group in late March 2020, there were often reports of long delays, and others reported receiving no information at all (22%) or conflicting information (10%). Qualitative analysis indicated that many participants who felt they should have been classified as CEV were not. Fig. 2 contains additional data on the varied reactions to shielding classification and text communications. More than 50% of those allocated to the shielding group agreed that the shielding allocation and communications had made them feel supported by the government and cared about by clinicians. However, 73.4% of shielders also agreed/strongly agreed that CEV classification and communications had increased worries about health:

It made me feel like they actually knew I had serious health conditions and that they cared. [yet also] … worried as it kind of makes you realize how vulnerable you really are and how dangerous.… (Participant 136, 30–39 years old)

A similar percentage (73.0%) reported feeling more worried about their health as a result of not being allocated to shielding. Interviews identified that they felt at greater risk, but without individual support or guidance in managing that risk. There was a statistically significant difference (Fisher’s exact test, *P* = 0.016) in the proportion of non-shielders (55.8%) who felt ‘abandoned’ due to their risk grouping and subsequent communications compared with shielders (25.5%):

I haven’t felt supported by the government at all, either individually or as a key worker.… As a high-risk group, I feel we were a forgotten grey area. (Participant 10, 60–69 years old)

#### Ongoing communication: keeping informed and supported

Following the shielding classification, shielded participants received communications offering advice and support from a variety of sources, including clinicians, local council officials, volunteers, NHS and/or government. However, the quality and quantity of communication and support received varied. The majority of interviewees appreciated both the information and ‘not being forgotten’:

They [volunteer] cared … called me at least four times.… They were really helpful, and I felt like the person I was talking to genuinely cares and gave me all the information that was available … the ’phone calls really made a difference. (Participant 136, 30–39 years old)

More than 50% found individual government/NHS text messages supportive and a source of helpful information, although a minority felt they were too impersonal or uninformative. However, government briefings were widely criticized owing to a lack of shielding information:

I feel that as a group, shielders have been ignored in general during the 3 months of lockdown and have not really been involved in any of the discussions about shielding. (Forum, 60–69 years old)

#### Pandemic communications received as part of UK general population

Central government’s decisions and communications were generally considered by participants to be inconsistent and indecisive, leading to widespread distrust. Opinions on communications from the devolved governments, especially Scotland, were generally positive in terms of perceptions of clarity and transparency. The frequent government reports highlighting that mortality was largely among those with ‘underlying health conditions’ was found to exacerbate social and medical insecurity in some:

I think it began with [Prime Minister’s] assertion that only the elderly and those with underlying conditions would be adversely affected.… By stating this, over and over again, he minimized this part of society. A huge group of people who, from the very top, didn’t really matter; it was ‘only’ them, almost expendable. (Participant 29, 40–49 years old)

Table 2 contains further quotations on positive and negative communication experiences.

**Table 2:** Communication experiences

Type of communication	Positive communication experiences	Negative communication experiences
Communication with individuals	**Appreciation of regular communication by multiple methods** It was really good, because they [local council] also ’phoned and there was a helpline if you needed anything, and they’d ’phone every now and again to check you were OK.… It was just knowing it was there and if I did need it, it was just a phonecall away.… You get your letters as well, so you’re always being reminded that there’s something there. (Participant 66, 40–49 years old)	**Disorganization and delays of initial shielding communication** I wasn’t told until the beginning of May that I was high risk from the virus.… I knew they were getting letters and I thought, where’s my letter? … I thought, well maybe I’m not; maybe they’ve reviewed me and I’m not high risk, but it was odd, so I was shielding anyway … confusing … disorganized.… I really could have done with their help at the very beginning. (Participant 12, 40–49 years old)
Ongoing communication: keeping informed and supported	**Support and information from LUPUS UK** If it hadn’t been for updates from LUPUS UK and my local [LUPUS UK] branch, it would have been awful.… It was extremely reassuring, like five-star reassuring, and to know that the helpline was there if I needed it, just there, that security was very reassuring. (Participant 22, 60–69 years old)	**Lack of government communication** To be mentioned in briefing once every 3 months when you are the part of society most impacted by this and whose lives have changed the most is poor. Even if they had nothing else to say, no new information, any sort of acknowledgement that we exist would have been welcomed. (Participant 29, 40–49 years old)
Pandemic communication as part of UK general population	**Confidence owing to perceived consistency** Nicola Sturgeon …she’s been there every day throughout the whole pandemic, and that’s given me a real confidence.… She takes questions and she properly answers them; she’s just brilliant. I really feel we’ve had very good, concise and clear messages. (Participant 47, 50–59 years old)	**Perception of indecisiveness and inconsistency of government guidance** Because there’s been so much to-ing and fro-ing with decisions, so are we wearing masks or not, are we in lockdown or not lockdown, there was just not really this is what we’re doing and here are the reasons, and we’re all going to stick to it. It was a bit wishy-washy, I think, [so] not all that confident really, so I guess that you’re going to have to look after yourself and make the decisions for yourself. (Participant 108, 30–39 years old)

### Theme 3: the impact of the risk classification and shielding on multiple domains (identity, society, support and behavioural)

Being classified (or not) as CEV had wide-ranging effects on multiple domains of participants lives.

#### Identity

For many participants, the shielding classification provided medical and societal acknowledgement, and validation of the severity of their disease. However, the term ‘clinically extremely vulnerable’ was sometimes reported to have negative impacts on social and self-identity:

I had lupus, but it didn’t stop me working, running, living my life. Then lockdown happened, and I got all the shielding texts basically saying don’t go out or you’ll die.… It feels like my whole identity and life has been pulled from underneath me, that I’m now a different person, an ill person, worse, whose illness impacts on those I love. (Forum, 40–49 years old)

Conversely, many of those who did not receive the shielding classification, yet felt they met the criteria, expressed feelings of abandonment. For many, this was experienced as a further extension of the invalidation faced when struggling to obtain a diagnosis, understanding, support and care:

Nobody’s contacted me, nobody’s told me to shield.… I feel neglected.… I asked the receptionist, “Can I ask why I haven’t had a shielding letter?”, and she said, “Well, it’s only sick people that have the letter”, and I said, “So you don’t consider me sick?”, and she said, “Well, you haven’t got cancer, have you?” (Participant 28, 50–59 years old)

#### Society: inclusion *vs* social stigma

Participants generally reported greater local social inclusion and support during shielding/lockdown than pre-pandemic. There was a feeling of solidarity from the whole population being ‘in the same boat’ as the chronically diseased in their restricted lifestyles, and recognition of the seriousness of their diseases. However, there were sometimes also perceptions of social stigma, particularly feeling ‘blamed’ that lockdown restrictions for the whole population were to protect only the vulnerable:

It makes you feel like you’re a burden on society, … message that has been sent out by the government, that we’ve all gone into lockdown to help shielded and the old people.… We’re made to feel othered, we are something else … incredibly isolating. (Participant 10, 60–69 years old)

#### Support with employers and provisions

The shielding classification led to support for working from home and obtaining provisions. The measures were generally very well received, although one shielder felt her employer’s insistence she work from home owing to her classification was discriminatory.

The negative impact of not receiving a shielding classification was often considerable, particularly for keyworkers expected to work in high-risk health-care/school environments, despite feeling at very high risk from COVID-19:

I really felt it [classification] was unfair.… I’ve never felt as scared to be potentially going into that [work] situation.… I was in tears a lot … panic-type symptoms … felt like I was alone.… It’s just been such a trauma, and the lupus has been so bad from the stress. (Participant 47, 50–59 years old)

Table 3 contains further views of the impact of shielding on identity, society and support.

**Table 3:** Patient views on impact of shielding/not shielding on identity and the social and practical impact

Identity	**Discordance in self *vs* official view of risk and dislike of disease being publicly known** It's scary to see it in black and white that we are extremely vulnerable and high risk. I've had lupus for 30 years, and I've never considered myself to be sick and vulnerable, so receiving the letters and texts is quite difficult to deal with.… In addition, I feel cross that my condition has now been exposed to everyone. (Forum, 50–59 years old) **Dislike of the term ‘vulnerable’ and increased fear owing to strongly worded risk messaging** I don’t have any help with anything, and to then be told I’m extremely vulnerable and actually at risk of this and there’s the likelihood that I’ll die if I catch this, it’s just scary.… I hate that [vulnerable] term, it’s a terrible way to put it … so they use such strong words because they want people to take it in and it to resonate with people. (Participant 80, 20–29 years old)
Social	**Increased understanding of severity of disease** I think when you have a chronic illness that isn’t visible you can be regarded as a hypochondriac.… If I was permanently in a wheelchair, most people would realize there is a serious problem.… The fact that I had to shield really brought it home to most of my family and friends. Nearly everyone telephoned to check on me. I had messages on our village Facebook page offering help. (Participant 124, 70–79 years old) **Blamed for lockdown, ‘cast aside’ by wider society** According to a large proportion of people, lockdown was introduced because of us … a constant refrain of, ‘keep the vulnerable, that word again, inside and let us get on with our lives.’ This has made me so, so incredibly sad. I’ve worked hard to make sure I contribute to society.… Now I’m cast aside, seen as someone that needs to be locked away so as not to inconvenience everyone else. I find it hurtful that so many people see that as a logical and acceptable solution. It really upsets me. (Participant 29, 40–49 years old) **Increased local community cohesion** She [daughter] said she's going to remember lockdown as the year her neighbours became her family. It was such a small thing, but the impact was huge. We all felt the community spirit. (Forum, 40–49 years old)
Support: employment and provisions	**Difficulties and stress for those not meeting shielding criteria to obtain evidence for employers** I think whatever criteria the government set out for these letters somehow skipped over SLE as an extremely complicated autoimmune disease itself.… GP really wanted me to ‘shield’ for the 12 weeks as I’m very at risk, but because the criteria didn’t match she couldn’t give me the letter. Obviously, everyone knows to use their common sense and isolate at this time, but I needed this letter for work and really had to fight for it. (Forum, 40–49 years old) **Practical support for shopping, medications and evidence for employers appreciated** I think I probably got more support as it shows how serious and dangerous my situation is … priority slots for my shopping to be delivered … medication delivered … got the free food boxes delivered from the government.… I don't need to physically return to work. It now feels a lot easier. (Participant 136, 30–39 years old) **Disparate views on quality of government-provided food boxes** The food parcels were excellent considering what they had to do quickly and for the numbers needed. (Survey, 40–49 years old) The cheapest stuff.… And that again that message from the government is, is that’s what you’re worth, we can furlough people at 80% of their salary, I’m not knocking that, we can pay for this and that, and we can give you all a Nando’s voucher now, but you shielding people are just worth the world’s worst toilet roll and an onion the size of your head. (Participant 10, 60–69 years old)

#### Behavioural

**Fig. 3:**
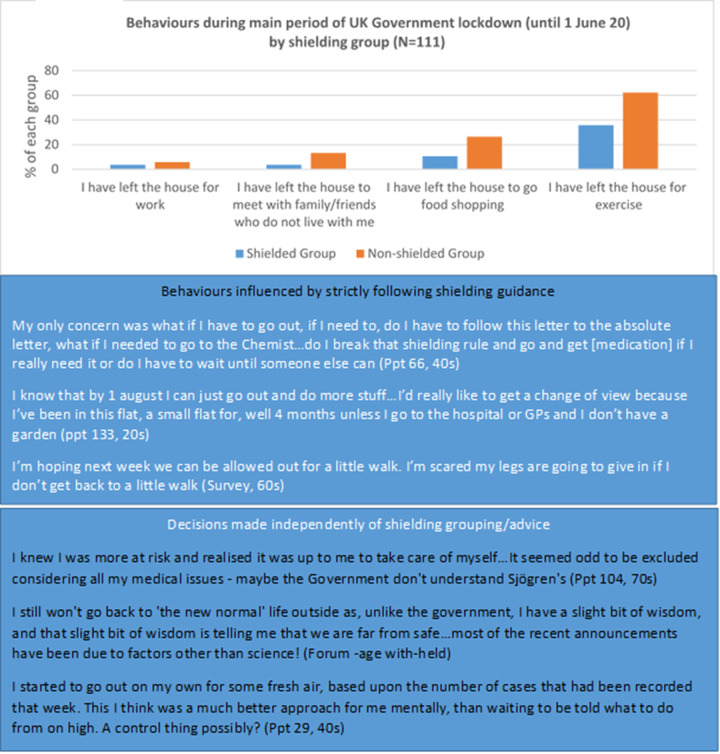
The range of behavioural choices made by participants

Risk classification and communications were reported (Fig. 2) by 84% of shielders (strongly agree/agree) to have changed their behaviour in terms of social mixing, compared with 52% of non-shielders (Fisher’s exact test on all five categories, *P* < 0.001). Discussions during interviews revealed that many participants were also balancing the risks and benefits themselves, with leaving the house for socially distant exercise being felt to be particularly important for their MH (Fig. 3). However, more non-shielders (62%) reported leaving the house for exercise as opposed to 36% of shielders (χ^2^ test, *P* = 0.006).

Many non-shielders decided to shield, and 71% of those not allocated to the CEV group agreed that they had made up their own minds regarding risk decisions, with the majority assessing themselves as higher risk than allocated:

I'm taking no chances. I haven't gone through all those years just to let some virus kill me because some Doc decides I don't score enough points. (Forum, 60–69 years old)

Distrust in government was discussed as reducing adherence to guidance, particularly in relationship to the planned relaxing of shielding/lockdown. Most participants felt this was politically/economically motivated and stated they would make their own decisions, usually involving maintenance of greater socially avoidant behaviour:

Even if the government say things are alright, I’m going to do what’s right for me … because they’ve not been truthful. (Participant 75, 40–49 years old)

### Theme 4: lessons from lockdown lifestyle for SARD management

In addition to the improvements in MH shown in Fig. 1, self-reported fatigue, pain and overall health scores all improved from pre-lockdown to during lockdown, with overall health (out of 100) improving from 46.67 to 50.59 (3.92, 95% CI: 0.24, 7.60, *P* = 0.030). This was explored in interviews, with some participants feeling that pacing was easier and less guilt inducing owing to more home-based and/or online work and social lives:

I don't need to physically return to work … offer to Zoom.… People now get that as a viable alternative, so that's a good outcome for me. (Participant 105, 50–59 years old)

Many participants discussed improvements in their health owing to the reduced pressure, both from themselves and from society, to maintain a normal life during lockdown:

I am in a much better place both mentally and physically than before lockdown.… I have been able to focus on me.… If I don’t feel like doing something … no guilt, shame, embarrassment. (Participant 111, 20–29 years old)

However, other participants reported increased symptoms and flares of disease activity. The importance of outdoor exercise was also regularly highlighted, and several considered that their health had worsened without the routine or purpose of work/socializing/exercising outside the home:

My mobility has been hugely affected … very frustrated, angry and emotional … managed my disease better when I was leaving the house; it gave me some purpose. Staying at home has left me alone with my pain. (Participant 132, 40–49 years old)

## Discussion

The principle of shielding was very well supported by the lupus and related SARD patients in this study. Many perceived it as a beneficent act of protection and care, providing information and practical support, and validation of the seriousness of their disease. However, the term ‘vulnerable’ was widely disliked. Some participants felt that being labelled ‘vulnerable’ subsumed all their other identities, exposed their disease status more widely than preferred and/or classed them as a separate (sometimes perceived as of lesser value) entity to the ‘normal’ population. This sense of being ‘othered’ [22] was reported by participants to have been exacerbated by government briefings often failing to inform or acknowledge the CEV group; providing reassurance to the general public that most deaths were among those with underlying health conditions [23]; and/or reports of the de-prioritization of people with such conditions in intensive care units [24]. This highlights the importance of communication strategies encouraging social cohesion and avoiding the implicit or accidental stigmatizing of any ‘vulnerable’ group.

Although strongly worded messaging about COVID-19 risk engendered more socially avoidant behaviours, it also greatly increased anxiety, which was reported by some to have precipitated disease flares. Fears were compounded by widespread distrust in central government, with perceptions of inconsistent messaging (although the devolved governments’ pandemic communications were generally viewed favourably) and rapidly changing guidance, as reported in other studies [25]. Although generating anxiety can ensure higher compliance with safety measures in pandemics [26], we agree with a recent call for encouraging adherence to behavioural guidance while also promoting wellbeing and ‘minimizing distress’ [27]. This might be helped by more personalized risk assessments and more frequent, less negatively framed messaging. Many participants perceived their mortality risk from COVID-19 as very high, when evolving understanding (rarely communicated to patients) was that it was much less than initially assumed and only marginally raised for most SARD patients [28, 29]. Although a recent study found a (slightly) increased all-cause mortality rate in March–April 2020 for patients with SARDs and similar diseases compared with the general population [30], it has not yet been ascertained how much of this increase was directly from COVID-19 or from the severe reduction and delays in medical care for lupus/SARDs, as reported in our previous study [14] and others [31].

Absent, delayed or miscommunication regarding shielding was identified in our research and other reports [32], and caused additional distress and anxiety. Although personalized contact was most appreciated, the largely positive views of generic NHS/government texts (examples in Supplementary Data S2, available at *Rheumatology Advances in Practice* online) suggest that these types of communication can be a cost-effective means of showing support and influencing behaviour (consistent with findings for other patient groups [33]) and could be developed further for post-pandemic times. Both non-shielders and shielders who perceived a lack of communication/support from clinicians or government used terminology suggestive of an overall feeling of abandonment, as found in other studies including SARDs patients [14, 15]. Feelings of abandonment were highest in non-shielders, who often felt that they should have been allocated to the shielding group. A study of patients with IBD also found a discordance between self-assessment and official COVID risk assessment, especially regarding immunosuppression [34].

Caution is required in research comparing officially allocated shielding/non-shielding groups (e.g. Kipps *et al.* [35]), because we found that shielding allocation did not lead to two distinct groups, owing to participants often making their own decisions. Although socially distant behaviour was reported to be greater in the government-allocated shielded group, most participants also balanced the risks and benefits themselves, particularly in terms of the improvements to MH from leaving the house for socially distant exercise [36]. The reduction in mobility and fitness reported by some shielding participants owing to confinement to the house was also found in studies of patients shielding because of other diseases [37]. Many non-shielders independently attempted to follow the shielding guidance, but were sometimes unable to do so without support with employment or provisions. This caused great stress and potentially increased risk of COVID-19, especially among some keyworkers who were expected to continue working. Being omitted from shielding classification/communication often reinforced medical insecurity, invalidation and the perception of lupus and related SARDs being widely misunderstood, generated from previous dismissive responses and misdiagnoses common in this patient group [12, 38, 39].

Overall wellbeing as measured by the WEMBS (<40 at both time points) places this cohort in the bottom 15% of UK population samples for wellbeing [17] and points to the requirement for more wellbeing support for SARD patients regardless of the additional pandemic challenges. There was an unexpected slight improvement (non-significant) between pre-lockdown WEMBS scores in early March 2020 and June 2020, and statistically significant improvements to multiple other health and wellbeing measures, including reductions in guilt, feeling a burden, and the impact of fatigue and pain on lives. Although not negating the need for urgent MH support for those most adversely impacted by the pandemic, this does raise questions regarding why this improvement might have occurred. Patients and the research team speculate that it might be attributable to the lockdown creating reduced pressure and greater opportunity to rest and pace activities without guilt or fear of societal or self-censure for being unable to participate fully in life. SARD patients might also have had greater resilience to potential adverse impacts on MH from lockdown/shielding as a result of having pre-adapted to a physically and socially constrained lifestyle and having more developed coping strategies than the general population. However, some situations that led to improvements are clearly not replicable in non-pandemic times, such as the reported reductions of sadness in missing out on ‘normal’ life owing to the general population being in lockdown.

Limitations of this study include the fact that diagnoses were self-reported. The very few male participants and an under-representation of some ethnic groups, as is common in research in this patient group [40], reduce the generalizabilty. Purposive sampling allowed for a more representative balance of ethnic groups for interviews, and other socio-demographic groups were well represented in both surveys and interviews. All participants were initially recruited through online support groups, and group members might differ in their experiences and behaviours compared with the wider lupus and SARD population. It is important to note that the findings of this study are from only the early stage of the pandemic, and views and wellbeing might well change as the pandemic progresses. Strengths include increased validation from triangulation from multiple data sources, member checking [20] and anomalous case analyses [21].

In conclusion, there was a widespread fear of dying from COVID-19 and a high level of support for social distancing measures, including shielding. Classification and communications were perceived as inconsistent, and many SARD patients who felt that they were at high risk from COVID-19 were not advised that they should shield. Although fear, isolation and the reduction in medical care were reported to have impacted physical and mental health negatively, some improvements occurred, probably attributable to a reduced pace of life during lockdown. This highlights the importance of clinicians advising on modifications to busy pre-disease lifestyles, self-management with pacing strategies and flexible working for many with chronic diseases where fatigue is a major life-changing symptom.

At the time of writing, ‘long COVID’ clinics are being established for those experiencing ongoing symptoms, including fatigue, cognitive dysfunction and pain. We strongly advocate for this type of support to be extended to patients with SARDs and other chronic diseases with similar debilitating symptoms that are often experienced for many years, with limited/no support to date.

## Supplementary data

Supplementary data are available at *Rheumatology Advances in Practice* online.

## Supplementary Material

rkab003_Supplementary_Data
