# Supplementary material for: COVID-19 and shielding: experiences of UK patients with lupus and related diseases
Source: Rheumatol Adv Pract. 2021 Jan 21;5(1):rkab003. doi: 10.1093/rap/rkab003 (PMC7928599; doi:10.1093/rap/rkab003)
Supplement: rkab003_Supplementary_Data [file rkab003_supplementary_data.docx]

**Supplementary Data S1: COREQ (COnsolidated criteria for REporting Qualitative research) Checklist^16^ and further methodology**

| Topic | Item No | Guide Questions/Description | Details and/or Reported on Page No. |
| --- | --- | --- | --- |
| **Domain 1: Research team and reflexivity** |  |  |  |
| *Personal characteristics* |  |  |  |
| Interviewer/facilitator | 1 | Which author/s conducted the interview or focus group? | MS, Page 5 |
| Credentials | 2 | What were the researcher’s credentials? | Page 5 + Supplementary Information |
| Occupation | 3 | What was their occupation at the time of the study? | Supplementary Information |
| Gender | 4 | Was the researcher male or female? | F, page 5 |
| Experience and training | 5 | What experience or training did the researcher have? | Supplementary Information |
| Relationship established | 6 | Was a relationship established prior to study commencement? | Supplementary Information |
| Participant knowledge of the interviewer | 7 | What did the participants know about the researcher? | Supplementary Information |
| Interviewer characteristics | 8 | What characteristics were reported about the interviewer/facilitator? | Supplementary Information |
| **Domain 2: Study design** |  |  |  |
| *Theoretical framework* |  |  |  |
| Methodological orientation and Theory | 9 | What methodological orientation was stated to underpin the study? | Page 4-5 supplementary information |
| *Participant selection* |  |  |  |
| Sampling | 10 | How were participants selected? | Page 4 + Supplementary Information |
| Method of approach | 11 | How were participants approached | Email  Supplementary Information |
| Sample size | 12 | How many participants were in the study | 25 (for interviews  Page 4 + supplementary information |
| Non-participation | 13 | How many people refused to participate or dropped out? | None refused but several uncontactable  Supplementary Information |
| *Setting* |  |  |  |
| Setting of data collection | 14 | Where was the data collected? | Supplementary Information |
| Presence of non-participants | 15 | Was anyone else present besides the participants and researchers? | Supplementary Information |
| Description of sample | 16 | What are the important characteristics of the sample? | Page 5 + supplementary information |
| *Data collection* |  |  |  |
| Interview guide | 17 | Were questions, prompts, guides provided by the authors? | Yes. Supplementary Information (Guide at supplementary information 1) |
| Repeat interviews | 18 | Were repeat interviews carried out? | Supplementary Information |
| Audio/visual recording | 19 | Did the research use audio or visual recording to collect the data? | Audio. Supplementary information |
| Field notes | 20 | Were ﬁeld notes made during and/or after the interview or focus group? | Yes. Supplementary Information |
| Duration | 21 | What was the duration of the inter views or focus group? | Approx 60 mins each. Supplementary information |
| Data saturation | 22 | Was data saturation discussed | Yes. Pg 5+ supplementary information |
| Transcripts returned | 23 | Were transcripts returned to participants for comment and/or correction? | No, but ¼ received the draft paper for approval of themes |
| **Domain 3: analysis and findings** |  |  |  |
| *Data analysis* |  |  |  |
| Number of data coders | 24 | How many data coders coded the data? | Two. Pg5 + supplementary information |
| Description of the coding tree | 25 | Did authors provide a description of the coding tree? | Available on request |
| Derivation of themes | 26 | Were themes identiﬁed in advance or derived from the data? | Derived from data. |
| Software | 27 | What software, if applicable, was used to manage the data? | Nvivo 12. Supplementary information |
| Participant checking | 28 | Did participants provide feedback on the ﬁndings? | Yes. Supplementary information |
| *Reporting* |  |  |  |
| Quotations presented | 29 | Were participant quotations presented to illustrate the themes/ﬁndings? | Yes. Supplementary Information |
| Data and findings consistent | 30 | Was there consistency between the data presented and the ﬁndings? | Yes. |
| Clarity of major themes | 31 | Were major themes clearly presented in the ﬁndings? | Yes. |
| Clarity of minor themes | 32 | Is there a description of diverse cases or discussion of minor themes? | Yes. |

**Domain 1: Research team and reflexivity**

The study team was multidisciplinary and included: rheumatologists, patients, behavioural scientists and data experts. This ensured a broad-range of experiences and expertise, with the differing perspectives reducing the chance of individual bias influencing the study. MS, an experienced, qualitatively trained, female research associate, carried out all interviews. Participants were fully informed about the research objectives and were given the opportunity to ask questions prior to both the surveys and the interviews. Participants were informed that the interviewer and several of the research team also had a SARD.

The study team and interviewer were known on the LUPUS UK forum and Facebook groups from previous studies. Information about the study and contact details were posted prior to recruitment.

**Domain 2: Study design**

This study was designed using mixed methods to allow for the qualitative elements to both explore and attempt to further explain^41^ the quantitative data received from the survey. This enhances the strengths from both methods, gives the human aspect behind the numbers/ statistics, and mitigates the weaknesses that can arise from analysing purely qualitative or quantitative data.

The study used a moderate realist^42^ and constructivist^43^ paradigm for the interviews. Researchers and interviewees therefore co-construct arising themes, with resulting overall themes combined from multiple interviews and incorporating realities that exist outside the researchers and interviewees own interpretations. This provides greater depth in understanding and the potential for identifying actions that may improve future experiences. Thematic analysis was used to generate initial themes from the first batch of interviews, with subsequent interviews then analysed using the constant comparative method, and this inductive-deductive analysis continuing with further interviews until the point that a reasonable degree of theoretical saturation had been achieved. Theoretical saturation occurred when subsequent interviews did not generate any novel codes or insights into the research question. Qualitative data was also collected from open-ended questions on the follow-up survey and analysis of pandemic-related questions on the LUPUS UK forum.

The participants in this study were from a cohort of SARDs participants who had joined a randomised controlled trial investigating the acceptability and effectiveness of small group email peer support and also provided the data for our study into the impact of COVID-19 on medical care^14^. Sign-up to the study was self-selected via the LUPUS UK online forum (>25,000 members) and Lupus support UK Facebook group (>7000 members). Analysis of the between-group differences from baseline-follow-up showed no significant differences in the primary outcome measure of the WEMBS so the data from both control and interventions groups could be included for the COVID-SARD sub-studies, as it was unaffected by the intervention.

**The study stages (*with key UK pandemic dates in italics*) are below:**

1. Baseline survey – completed online by 139 participants from 4-10 March 2020
2. *First UK COVID death reported on 5^th^ March*
3. *UK COVID Strategy announced on 5^th^ March: Contain. Delay. Research. Mitigate*
4. *COVID-19 declared a pandemic by WHO on 11 March 2020*
5. *'Clinical guide for the management of Rheumatology patients during the coronavirus pandemic' published by NHS England on 19^th^ March*
6. *BSR Guidance on COVID risk assessment relating to immunosuppression published on 22^nd^ March*
7. *Implementation of shielding for CEV’s announced on 22^nd^ March (estimated number 1.5 million)*
8. *UK lockdown commenced on 23 March 2020*
9. *UK peak of COVID fatalities reported as 8^th^ April*
10. Follow-up survey - completed online by 111 participants from 10-21 June 2020 during the pandemic.
11. Ethnographic immersion of patient-researchers in the LUPUS UK forum, March-July 2020
12. In-depth interviews with 25 purposively sampled survey participants – July 2020

Surveys assessed multiple measures of wellbeing/ mental health (MH), physical health and care, and incorporated various validated tools including the Warwick-Edinburgh Mental Wellbeing Scale (WEMWS)^17^. The WEMWS has 14 wellbeing categories with items such as optimism for the future, feeling close to other people and feeling confident. Participants are asked to assess each wellbeing item over the past 2 weeks, scoring on a scale of 1-5 with 1 being none of the time to 5 being all of the time. The range of scores are 14 (lowest wellbeing) to 70. In order to explore the impact of the pandemic and the shielding guidance, additional questions were included in the follow-up questionnaire. These included questions on reactions to COVID-19, shielding, changes to medical care and behaviours.

Following initial analysis of the quantitative data and early qualitative data, purposive sampling was used to select interviewees from survey responses. Selection was carried out by giving participants scores on level of level of positivity/negatively and MH scores, and ensuring the full range were interviewed whilst also ensuring a balance and range of key socio-demographic and disease characteristics such as age, ethnicity and length of time with the disease and an approximately equal proportion of shielders/ non-shielders in-line with survey proportions. Consent to be approached for interview was given at the end of the follow-up survey. Email addresses were given by those consenting and MS contacted potential interviewees by email to ascertain continued willingness to participate. Although no survey participants explicitly then declined to participate in the interviews, five did not respond to emails and two had interviews arranged but then were non-responsive when contacted. Interviews were mainly conducted on the telephone. Due to the success of our previous studies using email interviews several interviews were carried out by email if this was the interviewees preference. Probing questions to responses were then asked by follow-on emails.

Following input from all interested parties (patients, rheumatologist, LUPUS UK and psychologists) the interview schedule was designed to explore the impact of shielding. Interviews were semi-structured, with the interviewer asking key open-ended questions then following each participant’s priorities for discussion as long as they remained within the overall study direction, and gently re-directing when too much deviation from the aims occurred. Interviews utilised a mixture of standard questions (e.g. ‘What did you feel when you received your risk allocation?’) with each individual participant having additional specific questions to further probe further (e.g. ‘You said in your survey that receiving the texts made you worry more, please can you explain a bit more about why you think that was?’)

**Domain 3: analysis and findings**

The stages of analysis of qualitative data involved:

1) Ongoing researcher immersion in the data – This involved reading and re-reading transcripts/ communications/ forum posts throughout the study period in order to gain familiarity and depth of understanding.

2) Coding (classification) and re-coding the data –The coding scheme was designed to cover every aspect of the data, agreed by MS and RH, with MS coding all data and RH coding 25% (both using NVivo12). Forum posts, qualitative data from surveys and interview transcripts were combined at this stage for analysis. Any differences in coding were discussed and resolved, with input from MAB, and a new coding frame developed to reflect additional and combined codes.

3) Initial Identification of themes - Nvivo 12 was used to combine extracts from each transcript within each code. Both the coded data and raw data were viewed and discussed in-depth by MS and patient- researchers. Individual codes were then combined as emerging themes became clearer from both the data and discussions. EL, FN, CG, MAB, MB and DDC were provided with the raw data. MAB and EL carried out an independent analysis of the coded data in order to ensure united decisions on which were key emerging themes and thus reduce the potential for individual researcher bias in the generation and prioritisation of themes.

4) Common themes leading to more abstract concepts were then discussed and agreed by the research team. Due to the political nature of this topic, divergent views were expressed on the level of inclusion of the more contentious topics regarding Government, especially the comparison between central and devolved governments. It was decided that accurately representing participant views was the priority with the caveat that these were participant views rather than the views of the authors or LUPUS UK.

5) A random selection of interviewees were sent the draft paper with themes for member checking, and their input considered in later drafts.

Key findings were illustrated by the use of participant quotes. These were anonymised, using only participant number and age.

With regards to the quantitative data, only those completing both baseline and follow-up surveys were included in this sub-study (111 out of 139 completing baseline – an 80% response rate). There was no evidence of bias between characteristics of non-responders/responders with the exception of a higher follow-up rate among younger (<30 years) participants. In terms of mental and physical health, there were differences between the 111 participants who completed follow-up and the 28 who were lost to follow-up, with those lost to follow-up self-assessing as generally having overall poorer health. For example, the overall health score (out of 100) on the day of completing the baseline questionnaire was a mean of 41.0 for non-responders at follow-up and 46.7 for responders (-5.6, 95%CI -14.7 to 3.4, p=0.222) . This attrition may have caused an under-estimation of any effects (baseline - follow-up) being measured in this study.

**Supplementary Data S2: Example text messages received by the shielding group from ‘NHS Coronavirus Service’**

| We have identified that you’re someone at risk of severe illness if you catch Coronavirus. Please remain at home for a minimum of 12 weeks. Home is the safest place for you. Staying in helps you stay well and that will help the NHS too. You can open a window but do not leave your home, and stay 3 steps away from others indoors. Wash your hands more often, for at least 20 seconds… |
| --- |
| Do you live with others/ This advice will keep you safer from the virus:  -Sleep separately if you can  -Stay 3 steps away from others at home  -Keep away from children  -Only essential carers should visit  - wash your hands more often for 20 seconds and always before eating. Moisturise if your skin gets dry  - Eat separately, using your own cutlery, dishcloth and towels  -If you can, use separate bathrooms. If you share a bathroom, use it first and clean between uses |
| Check you have a few important items for the next few weeks such as frozen or tinned vegetables, dry food and soap for handwashing. Don’t worry if you’re missing any items. Ask a friend, neighbour or relative to leave what you need at your door. If you need support, use [link] to ask for help.  Be prepared in case you need to go to hospital, get a single bag ready with your key health information and essentials for an overnight stay. The latest advice is here [link] |
| Keep moving to stay healthy  -do some simple stretches and walk around  - Be kind to yourself  - spend time doing things you enjoy- reading, cooking and other indoor hobbies  -Try to eat healthy, well-balanced meals, and drink enough water  - Spending time with the windows open to let in fresh air, sit and look out at the world where you can, or get out into your garden or sit on your doorstep if you can.  To opt out reply STOP |
